# Influence of cell cycle on responses of MCF-7 cells to benzo[a]pyrene

**DOI:** 10.1186/1471-2164-12-333

**Published:** 2011-06-29

**Authors:** Hamza Hamouchene, Volker M Arlt, Ian Giddings, David H Phillips

**Affiliations:** 1 Section of Molecular Carcinogenesis, Institute of Cancer Research, Brookes Lawley Building, Cotswold Road, Sutton, Surrey SM2 5NG, UK

## Abstract

**Background:**

Benzo[a]pyrene (BaP) is a widespread environmental genotoxic carcinogen that damages DNA by forming adducts. This damage along with activation of the aryl hydrocarbon receptor (AHR) induces complex transcriptional responses in cells. To investigate whether human cells are more susceptible to BaP in a particular phase of the cell cycle, synchronised breast carcinoma MCF-7 cells were exposed to BaP. Cell cycle progression was analysed by flow cytometry, DNA adduct formation was assessed by ^32^P-postlabeling analysis, microarrays of 44K human genome-wide oligos and RT-PCR were used to detect gene expression (mRNA) changes and Western blotting was performed to determine the expression of some proteins, including cytochrome P450 (CYP) 1A1 and CYP1B1, which are involved in BaP metabolism.

**Results:**

Following BaP exposure, cells evaded *G1 *arrest and accumulated in *S*-phase. Higher levels of DNA damage occurred in *S*- and *G2/M*- compared with *G0/G1-*enriched cultures. Genes that were found to have altered expression included those involved in xenobiotic metabolism, apoptosis, cell cycle regulation and DNA repair. Gene ontology and pathway analysis showed the involvement of various signalling pathways in response to BaP exposure, such as the Catenin/Wnt pathway in *G1*, the ERK pathway in *G1 *and *S*, the Nrf2 pathway in *S *and *G2/M *and the Akt pathway in *G2/M*. An important finding was that higher levels of DNA damage in *S- *and *G2/M*-enriched cultures correlated with higher levels of *CYP1A1 *and *CYP1B1 *mRNA and proteins. Moreover, exposure of synchronised MCF-7 cells to BaP-7,8-diol-9,10-epoxide (BPDE), the ultimate carcinogenic metabolite of BaP, did not result in significant changes in DNA adduct levels at different phases of the cell cycle.

**Conclusions:**

This study characterised the complex gene response to BaP in MCF-7 cells and revealed a strong correlation between the varying efficiency of BaP metabolism and DNA damage in different phases of the cell cycle. Our results suggest that growth kinetics within a target-cell population may be important determinants of susceptibility and response to a genotoxic agent.

## Background

Chemical carcinogens that act by a genotoxic mechanism exert their biological effects through damaging DNA. This damage can be manifested in several forms, including single or double strand breaks, apurinic sites and covalent modification of the bases. Some chemical carcinogens such as benzo[a]pyrene (BaP), which is a representative of the class of polycyclic aromatic hydrocarbons (PAHs), are thought to cause cancer through covalent binding of their reactive metabolites to DNA, forming DNA adducts [1-3]. BaP-7,8-diol-9,10-epoxides (BPDE), the ultimate carcinogenic metabolites of BaP, react predominantly with the N^2 ^position of guanine residues and to a lesser extent with the N^6 ^position of adenine residues in DNA [4].

In mammalian cells BaP binds to the aryl hydrocarbon receptor (AHR), which is a cytosolic ligand-activated transcription factor that functions as a sensor of extracellular signals and environmental stresses affecting cell growth and development. AHR controls the expression of genes coding for xenobiotic-metabolising enzymes such as cytochrome P450s (CYPs), UDP-glucuronosyltransferase UGT1A6, NAD(P)H:quinone oxidoreductase-1 (NQO1), aldehyde dehydrogenase (ALDH3A1), and several glutathione-S-transferases [5]. It is also involved in regulation of development and in the control of circadian rhythms, neurogenesis and stress response to hypoxia [6].

More recently it has also become evident that AHR has another function, namely in controlling cell cycle progression. For instance, high-affinity AHR ligands, such as some PAHs, cause a wide range of cell-cycle perturbations, including *G0/G1 *arrest or its evasion (the stealth property) [7], *G2/M *arrest, *S-*phase accumulation, diminished capacity for DNA replication and inhibition of cell proliferation [8]. These perturbations have been documented in several gene expression profiling studies. Previously we have used microarray technology to analyse the transcriptomes of several human cell lines exposed to BaP [9,10]. Altered expression of a number of genes involved in cell cycle regulation were identified, including *CDKN1A, MAK, BTG2, CCNG1 *and *E2F6*. Other studies have shown that up-regulated AHR-dependent activation of CYP1A1 following BaP exposure may be dependent on the cell-cycle phase [11-13], suggesting that the phase of the cell cycle may be critical to some of the effects of BaP on human cells.

In this study, we investigated whether cells are more susceptible to a genotoxic carcinogen, namely BaP, at particular phases of the cell cycle and, if so, to elucidate the processes involved. DNA microarrays were used to examine changes in gene expression throughout the cell cycle in synchronised human breast carcinoma MCF-7 cells following exposure to non-cytotoxic concentrations of BaP. Cell cycle phase-specific changes in gene expression profiles resulting from carcinogen exposure have identified novel genes and pathways potentially involved in the carcinogenic process. To strengthen the process of identifying target genes, gene expression data were compared to other biological parameters, including DNA adduct formation, determined by ^32^P-postlabelling analysis, and cell cycle progression, measured by FACS analysis.

## Results

### Cell cycle progression

In initial experiments, the optimum time of treatment with BaP was determined to be 12 h. This gave sufficient time for cells to metabolise BaP to DNA-binding reactive intermediates, but minimised the extent to which untreated synchronised cells altered their cell-cycle phase composition. In the case of *G0/G1 *enrichment, cells will start exiting the quiescent state (*G0) *and entering *G1 *soon after adding the serum back to the medium. Thus, from now on, these cells are referred to as *G1*-enriched. In previous work, the treatment concentration of 2.5 μM was found to induce DNA adduct formation in MCF-7 cells within a linear dose-response range [9].

**Figure 1:**
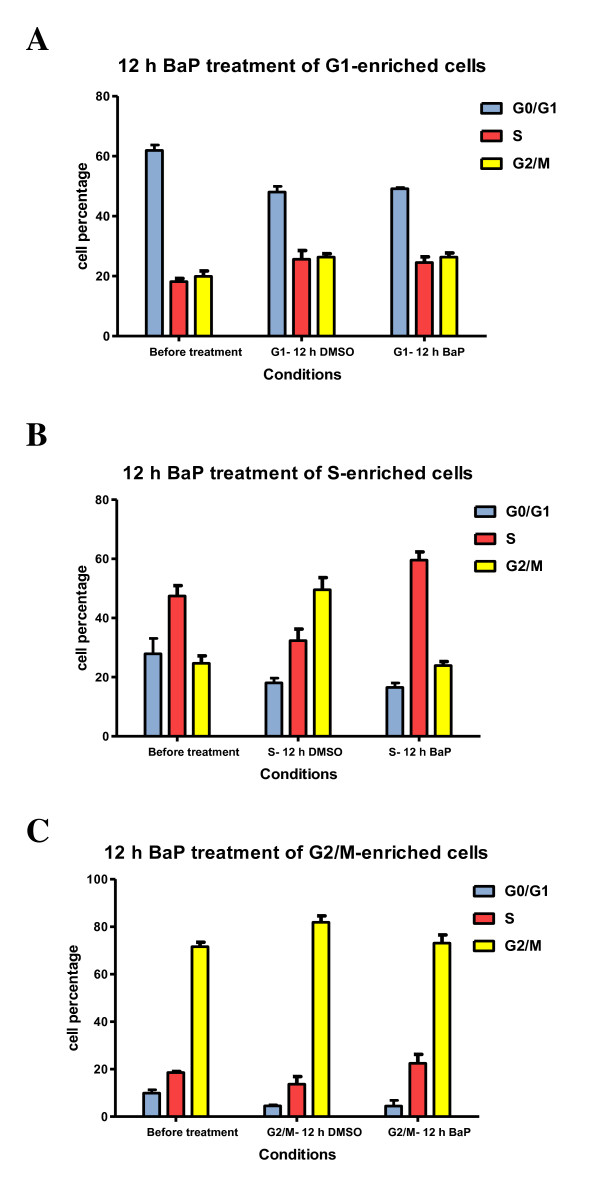
**BaP delays escape from S-phase**. **A**- MCF-7 cells were synchronised in *G0/G1*-phase by serum-deprivation for 48 hours, after which cells were exposed for 12 h to BaP (2.5 μM) or DMSO. **B**- MCF-7 cells were enriched in *S*-phase by serum-deprivation for 48 h, then left to grow for 18 h, after which they were treated by either BaP (2.5 μM) or DMSO for 12 h. **C**- MCF-7 cells were synchronised in *G2/M*-phase by exposing them to aphidicolin (1 μg/mL) for 24 h followed by colchicine (0.25 μM) for 12 h. Subsequently, they were released into media containing either BaP (2.5 μM) or DMSO for 12 h. Cell cycle distribution was examined by flow cytometry. The profiles are representative of three independent experiments.

*G1*-enriched cultures (serum deprivation for 48 h) did not differ significantly in the proportions of cells in different phases after treatment for 12 h with BaP compared with DMSO-treated controls (Figure 1A and Additional file 1A). Cells were progressing through the cell cycle and started entering *S *and *G2/M phases *by the end of the treatment. We did not observe a *G1 *arrest after BaP treatment.

Exposure of *S-*enriched cultures (18 h after 48 h of serum deprivation) to BaP evoked dramatic alterations in cell cycle distribution with an increase of the fraction of cells in *S*-phase (59.6 *vs *32.4, *P *< 0.001; Figure 1B and Additional file 1B). The percentage of cells in *G2/M-phase *was significantly lower than in control cultures (*P *< 0.001).

Similarly, BaP treatment of *G2/M*-enriched cultures (24 h treatment with 1 μg/mL aphidicolin followed by 12 h 0.25 μM colchicine) increased the proportion of cells in *S-*phase (17.6 *vs *11.2, *P *= 0.0045; Figure 1C and Additional file 1C).

### DNA damage in synchronised MCF-7 cells

**Figure 2:**
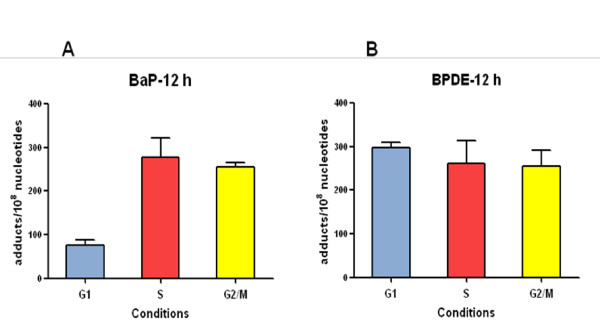
**DNA adduct levels in synchronised MCF-7 cells**. Cell were synchronised in *G0/G1, S *and *G2/M *phases with different methods, after which they were exposed to 2.5 μM BaP or 0.5 μM BPDE for 12 h. DNA was isolated with a standard phenol/chloroform method and DNA adducts were assessed by the ^32^P-postlabelling method. Results were expressed as DNA adducts/10^8 ^nucleotides.

BaP-DNA adduct formation was determined by the ^32^P-postlabelling method. Cells enriched in *G1*, *S *and *G2/M *that were exposed to BaP for 12 h showed different levels of DNA adducts (Figure 2A). Levels of adducts in the *S- *and *G2/M-*enriched cultures were 3 to 4-fold higher than levels observed in *G1*-enriched cultures.

When cells were treated with BPDE for 12 h, the reactive metabolite of BaP, similar levels of DNA adducts were formed in all cultures regardless of cell-cycle phase (Figure 2B). Since BPDE does not require metabolic activation to bind to DNA, and has a short half-life in aqueous environments, this result suggests that the differences observed with BaP are the consequence of different capacities to metabolically activate BaP at different stages of the cell cycle.

### BaP-induced gene expression changes by microarray analysis

cDNA microarray analysis was carried out on synchronised cultures of MCF-7 cells enriched in *G1*, *S *and *G2/M *phases and exposed to 2.5 μM BaP for 12 h.

**Figure 3:**
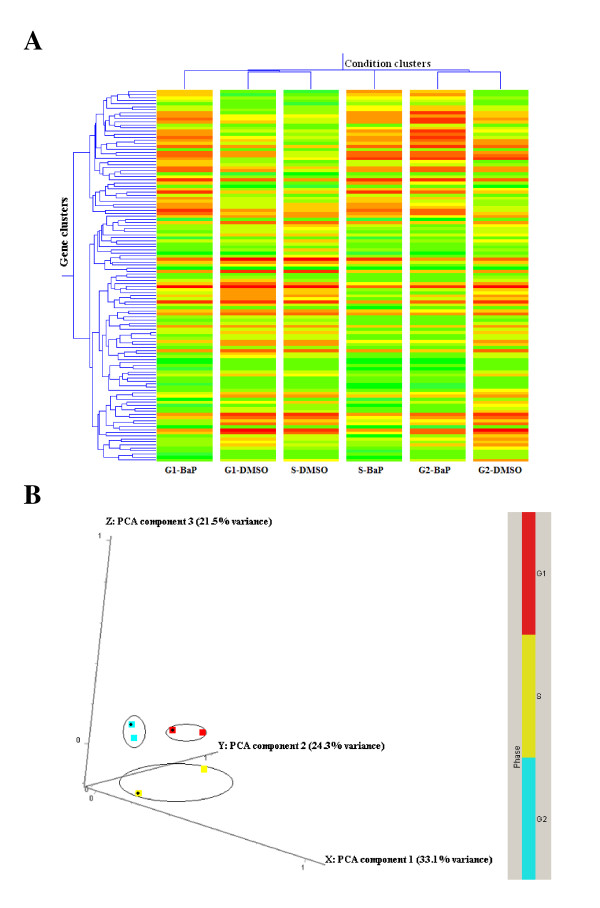
**Cell cycle effects on microarray results**. **A**- Hierarchical clustering of genes in different conditions; **B**- Principal component analysis (PCA). Both methods were performed in GeneSpring on a list of genes that had good confidence measurement and revealed a cell cycle response in gene expression profiles. Before BaP (2.5 μM) treatment for 12 h, MCF-7 cells were synchronised in different phases of the cell cycle. In hierarchical clustering (**A**), red colour denotes up-regulation and green denotes down-regulation. In PCA analysis (**B**), squares with black dots denote BaP-treated samples.

Condition clustering and principal component analysis (PCA) revealed that exposure to BaP resulted in expression profiles more distinguishable by cell-cycle phase than by treatment (Figure 3).

**Figure 4:**
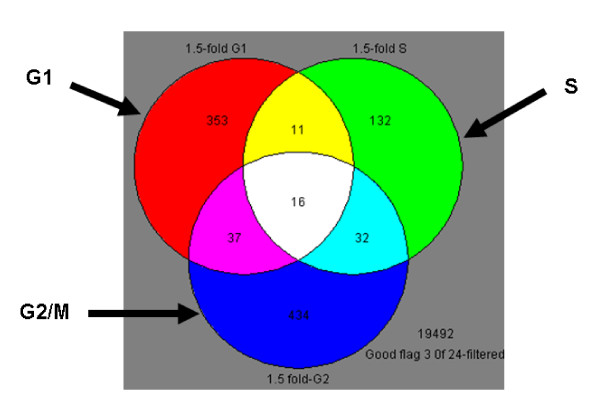
**Venn diagram of the gene lists in different phases**. Only genes that exhibited 1.5-fold or greater change after BaP treatment are shown. The gene lists represent expression profiles of MCF-7 cells synchronised in different phases of the cell cycle. Although there was overlap of the expression profiles between the phases, the majority of the changes were cell-cycle dependent.

Differentially expressed genes in each enriched culture were identified using Student's *t*-test and a cut-off of 1.5-fold change in expression. This resulted in 417 genes in *G1*-, 189 genes in *S*-, and 519 genes in *G2/M-*enriched cultures (Figure 4). 16 genes were shared between all phases (Additional file 2), 11 between *G1 *and *S *only (Additional file 3), 37 between *G1 *and *G2/M *only (Additional file 4), and 32 between *S *and *G2/M *only (Additional file 5). However, the majority of modulated genes were cell-cycle specific (Figure 4).

#### Functional annotations of BaP-modulated genes

In order to find biological processes significantly over-represented in the gene lists generated by statistical analysis, overlay of gene ontology information was carried out using the Gene Ontology (GO) function within GeneSpring. Biological themes that occurred in response to BaP through the cell cycle were thereby identified. The majority of functions identified indicate that the transcriptional response to BaP in MCF-7 cells in different phases is complex, with a large number of biochemical and molecular pathways being affected (Table 1).

**Table 1:** Biological processes significantly affected (p < 0.05) and over-represented after BaP treatment in synchronised MCF-7 cells in *G0/G1*, *S*, and *G2/M *as determined by Gene Ontology (GO) analysis within GeneSpring software

Biological Process	% of Genes in List	p-Value
***G1*-enriched cells**		

GO:7275: development	26.4	1.50E-03

GO:9653: morphogenesis	11.8	1.60E-02

GO:30154: cell differentiation	11.4	1.20E-02

GO:9059: macromolecule biosynthesis	10	1.10E-02

GO:8283: cell proliferation	8.6	1.90E-02

GO:31325: positive regulation of cellular metabolism	5	3.10E-02

GO:45941: positive regulation of transcription	4.1	4.90E-02

GO:45321: immune cell activation	3.2	2.30E-02

GO:51606: detection of stimulus	2.3	4.00E-03

***S*-enriched cells**		

GO:50789: regulation of biological process	42.7	1.30E-02

GO:42127: regulation of cell proliferation	7.3	2.10E-02

GO:40007: growth	5.5	1.70E-02

GO:42592: homeostasis	5.5	4.10E-02

GO:45944: positive regulation of transcription from RNA polymerase II promoter	3.6	2.20E-02

GO:7420: brain development	2.7	4.60E-02

GO:9065: glutamine family amino acid catabolism	1.8	7.20E-03

GO:6805: xenobiotic metabolism	1.8	2.10E-02

GO:9266: response to temperature stimulus	1.8	2.10E-02

GO:7157: heterophilic cell adhesion	1.8	2.20E-02

GO:6664: glycolipid metabolism	1.8	2.70E-02

GO:30203: glycosaminoglycan metabolism	1.8	5.00E-02

GO:35026: leading edge cell differentiation	0.9	5.60E-03

GO:42268: regulation of cytolysis	0.9	5.60E-03

GO:46399: glucuronate biosynthesis	0.9	1.10E-02

GO:16264: gap junction assembly	0.9	1.70E-02

GO:6975: DNA damage induced protein phosphorylation	0.9	1.70E-02

GO:48246: macrophage chemotaxis	0.9	3.30E-02

***G2/M*-enriched cells**		

GO:19219: regulation of nucleobase, nucleoside, nucleotide and nucleic acid metabolism	23.9	1.40E-03

GO:6355: regulation of transcription, DNA-dependent	21.8	1.80E-02

GO:30154: cell differentiation	10.6	1.10E-02

GO:7049: cell cycle	9.9	2.70E-04

GO:7264: small GTPase mediated signal transduction	6.3	6.90E-03

GO:9888: tissue development	4.2	1.30E-02

GO:16337: cell-cell adhesion	3.5	6.20E-03

GO:165: MAPKKK cascade	2.8	1.70E-02

GO:31098: stress-activated protein kinase signaling pathway	1.8	2.30E-03

GO:8624: induction of apoptosis by extracellular signals	1.4	3.70E-02

GO:42551: neuron maturation	1.4	3.00E-03

GO:6986: response to unfolded protein	1.4	1.10E-02

GO:51259: protein oligomerization	1.4	2.60E-02

GO:9266: response to temperature stimulus	1.1	1.50E-02

Genes over-represented had an expression change of at least 1.5-fold.

In *G1*, genes involved in macromolecule metabolism were over-represented by four functional groups: macromolecule biosynthesis (10%), positive regulation of metabolism (5%) and transcription (4%), and amino acid transport (2%). These genes are involved in RNA transcription and protein synthesis and code for several ribosomal proteins (for example *RPS10, RPS14*, and *RPS15A*), solute carriers (*SLC6A6, SLC7A11 *and *SLC6A14*), and regulators of transcription (*ATF4, JUN, EGR1, RSF *and *TRERF1*). Other modulated genes belonged to cell differentiation (11%) and cell proliferation (8%) functional groups.

In *S*-phase, cell proliferation functional groups (7%) were again identified including the genes *BTG2, BTG3, GAS8 *and *HDAC4*. Of these, *BTG2 *and *BTG3 *(both up-regulated) belong to a family of anti-proliferative genes. Genes involved in PAH metabolism were also over-represented and these included *CYP1B1*, *AKR1C1, ALDH1A3 *and *UGT1A6*.

In *G2/M*-phase, the largest functional groups identified were regulation of nucleic acid metabolism (24%) and regulation of transcription (22%), followed by cell differentiation (10%) and cell cycle (10%). Cell cycle regulation genes induced by BaP included *NPM1, NBN, FHIT, CABLES2, ATF5, PCAF, CCNG1, RGC32, SESN1 *and *BAX*. Signal transduction genes were represented by several functional groups such as small GTPase-mediated signal transduction (6%), MAPKKK cascade (3%) and stress-related protein kinase signalling pathway (2%).

#### Pathway analysis of BaP-modulated genes

**Figure 5:**
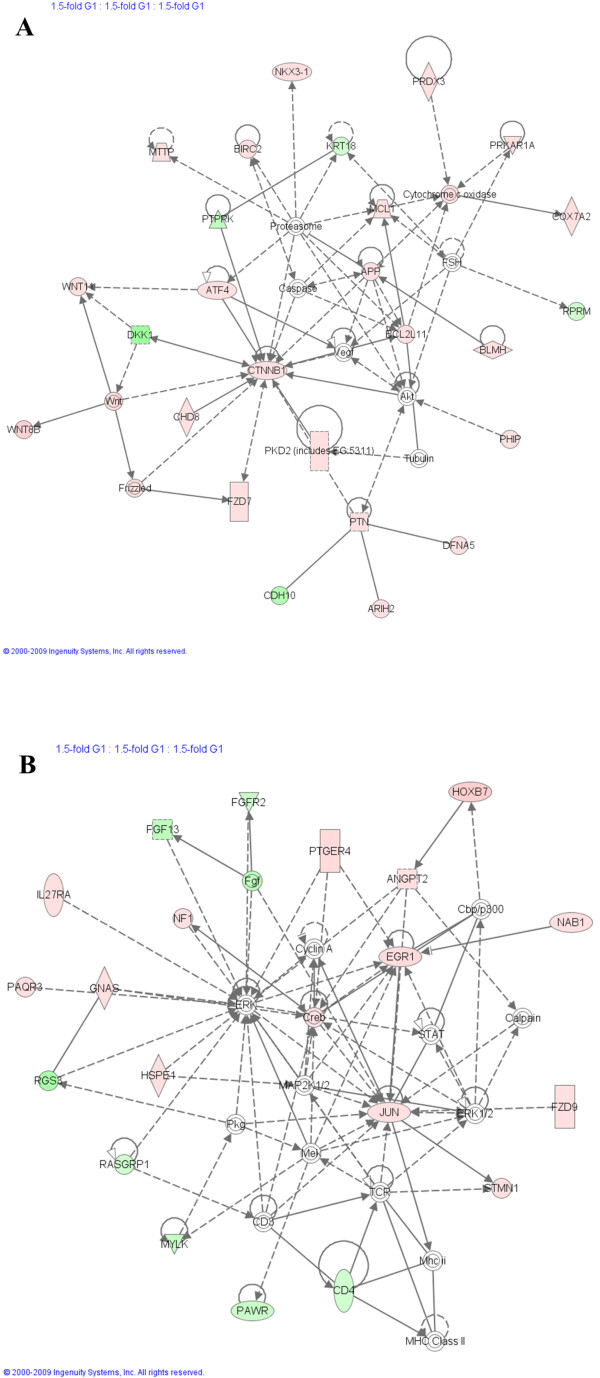
**Ingenuity Pathway Analysis (IPA) on genes modulated by BaP in *G1*-enriched MCF7 cell cultures**. Gene lists of 1.5-fold differentially expressed genes in different phases were imported to IPA software, which revealed the involvement of several pathways and genes in the response to BaP. Two networks are shown here. Red colour denotes up-regulation and green colour denotes down-regulation. The IPA legend is shown in **Additional file 6**.

**Figure 6:**
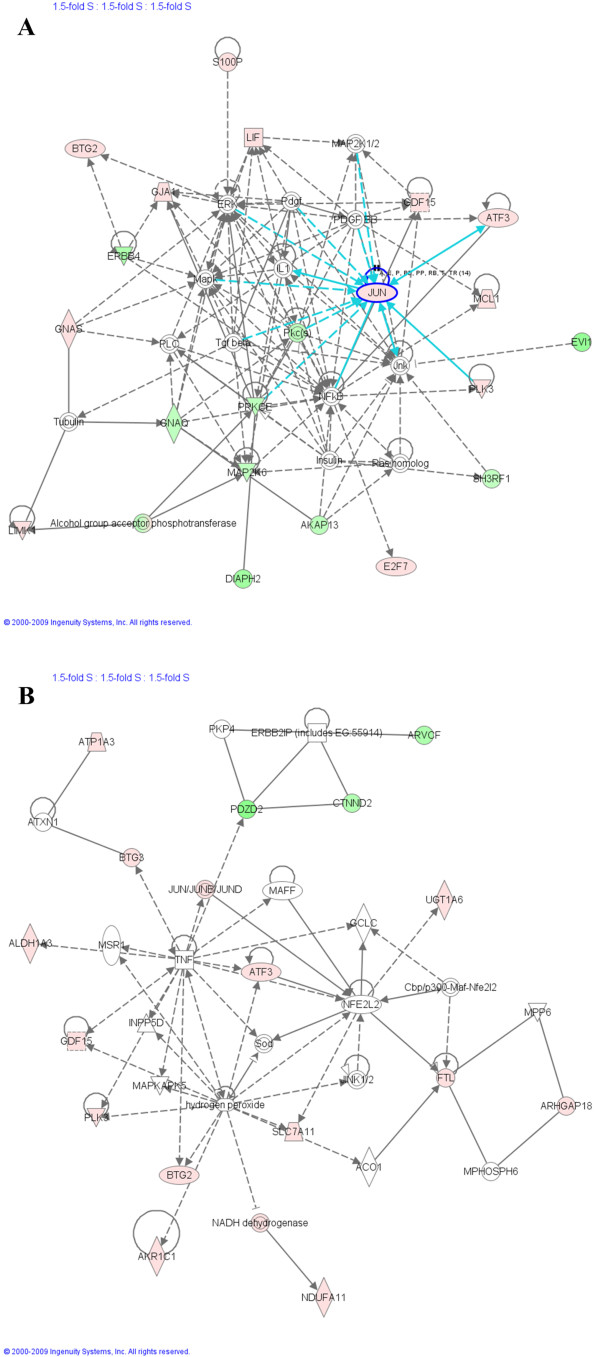
**Ingenuity Pathway Analysis (IPA) on genes modulated by BaP in *S-*enriched MCF-7 cell cultures**. Gene lists of 1.5-fold differentially expressed genes in different phases were imported to IPA software, which revealed the involvement of several pathways and genes in the response to BaP. Two networks are shown here. Red colour denotes up-regulation and green colour denotes down-regulation. The IPA legend is shown in **Additional file 6**.

**Figure 7:**
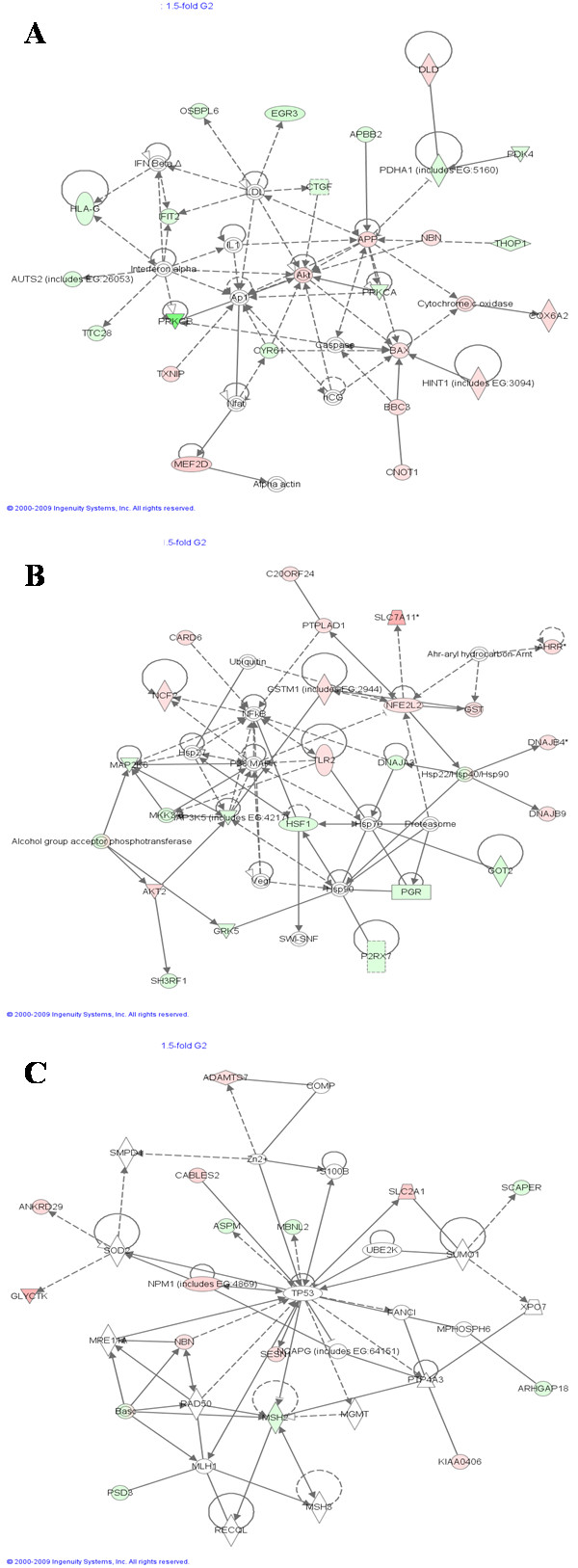
**Ingenuity Pathway Analysis (IPA) on genes modulated by BaP in *G2/M-*enriched MCF-7 cell cultures**. Gene lists of 1.5-fold differentially expressed genes in different phases were imported to IPA software, which revealed the involvement of several pathways and genes in the response to BaP. Three networks are shown here. Red colour denotes up-regulation and green colour denotes down-regulation. The IPA legend is shown in **Additional file 6**.

The generated gene lists from GeneSpring were submitted to Ingenuity Pathway Analysis (IPA) and several interesting pathways and genes were revealed. Seven selected networks are presented here (Figure 5, 6 and 7 with Additional file 6); they are the top two scoring networks in each enriched culture and a third one for *G2/M-*enriched cultures. This selection was based on the ranking score within IPA, where networks with the highest number of significant changes rank highest in the list (a list of the top five scoring networks in each enriched culture is presented as supplementary data in Additional file 7). Several biological processes and signalling pathways were shown to be at the centre of BaP-modulation, as described below.

##### *G1*-phase (Figure 5, Network A and B)

Network 5A relates to the Catenin/Wnt pathway, which has a crucial role in embryonic development. Its deregulation can induce disease, most importantly cancer. One important gene in this pathway is *CTNNB1*, which stabilises β-catenin, a cytoplasmic protein that translocates into the nucleus and activates downstream genes such as *MYC *and cyclin D1, both of which regulate cell proliferation [14].

Network 5B involves mainly the *JUN, EGR1 *and *ERK *pathway. *JUN*, which is up-regulated in both *G1*- and *S*-phases, is an oncogene and a transcription factor that plays a role in the regulation of normal cell cycle progression [15]. *EGR1 *is another transcription factor that was up-regulated at the mRNA level in our study. It is at the crossroads of many signalling cascades. Many functions have been attributed to this protein, in particular its involvement in the control of cell growth, survival and transformation [16].

##### *S*-phase (Figure 6, Network A and B)

*JUN *is again the focus of Network 6A and it is linked to several other genes, for example *GDF15 *and *ATF3*, both of which were up-regulated in the present study.

*ATF3 *is a member of the ATF/cyclic AMP response element-binding family of transcription factors. It has been proposed that it has a dichotomous role in cancer development by promoting or suppressing apoptosis and proliferation [17]. *GDF15 *is a member of the transforming growth factor B (*TGFB*) superfamily that regulates tissue differentiation and maintenance. It is also a transcriptional target of p53 [18]. Another interesting gene shown in this network is *NFκB*, which promotes cell survival.

Network 6B shows mainly molecules that are involved in oxidative stress; *NFE2L2 *(NRF2) is induced in response to reactive oxygen species such as hydrogen peroxide. *NRF2 *plays a major role in the protective mechanism against xenobiotics capable of damaging DNA and initiating carcinogenesis. It is a cellular sensor of chemical- and radiation-induced oxidative and electrophilic stress, and a nuclear transcription factor that controls the expression and coordinated induction of a battery of defensive genes encoding detoxifying enzymes and antioxidant proteins. One of these proteins is NQO1 [19], which is mentioned in the Introduction.

##### *G2/M*-phase (Figure 7, Network A, B and C)

Network 7A involves two pathways, Akt and apoptosis. Akt is a serine/threonine protein kinase that, when activated, plays a key role in mediating signals for cell growth, cell survival (anti-apoptotic), cell-cycle progression, differentiation, transcription, translation, and glucose metabolism. Recent advances in studying Akt signalling have uncovered important roles in *G2/M *transition of the cell cycle where Akt activity is highest [20]. The NRF2 pathway is again central to Network 7B, which shows several genes involved in oxidative stress mechanisms such as *NFE2L2*, *GSTM1*, *SLC7A11*, and *AHRR*.

Network 7C was selected to be shown here because it has the important tumour suppressor *TP53 *at its centre along with several of its targets, confirming results obtained by gene ontology analysis. *ASPM *participates in the normal mitotic spindle function while *MBNL2 *belongs to the muscleblind family that regulates alternative splicing. *CABLES2 *is a pro-apoptotic factor involved in p53-dependent or p53-independent apoptosis [21] while *Scaper *is a cyclin A-interacting protein that regulates cell cycle progression at the *G/S *and *G2/M *checkpoints [22].

### BaP-induced gene expression changes by RT-PCR

RT-PCR is a more sensitive and specific measure of gene expression and was used to validate a number of key expression changes and to determine the reliability of the microarrays. Genes were selected for RT-PCR validation on the basis of: a) GeneSpring statistical analysis, b) gene ontology analysis and c) pathway analysis.

**Figure 8:**
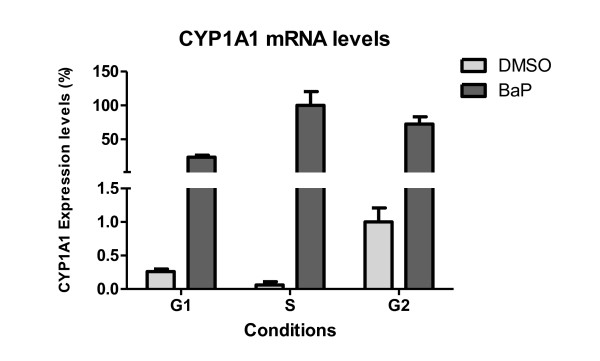
**Relative *CYP1A1 *mRNA expression levels in synchronised MCF7 cells with and without BaP treatment**. mRNA quantification was carried out by RT-PCR. The highest expressed sample in the RT-PCR was set to 100% and other samples' expressions are shown relative to that. Values represent mean ± SD from 3 determinations.

Genes validated by RT-PCR are shown in Table 2. In the majority of cases there was a good correlation between RT-PCR and microarray results, RT-PCR being more sensitive; expression ratios were generally underestimated by microarray analysis. For *CYP1A1*, the correlation between the two methods was very low; no clear change in this transcript was evident from the microarrays, whereas RT-PCR identified strong induction in all phases ranging from 74-fold in *G2/M-*enriched cultures to over 1800-fold in *S-*enriched cultures (Figure 8). The failure of the microarrays to identify this gene expression change may be a result of very low basal levels of this transcript in this cell line, such that even if strongly induced, the microarrays are not sensitive enough to detect it. Another explanation could be the quality and specificity of the probe sequence in the array.

**Table 2:** A summary of gene expression changes induced by BaP in MCF7 cells using microarray and RT-PCR analysis

		Change in *G1*		Change in *S*		Change in *G2/M*	
**Gene Symbol**	**Biological function**	**RT-PCR**	**Microarrays**	**RT-PCR**	**Microarrays**	**RT-PCR**	**Microarrays**

*CYP1A1*	Xenobiotic metabolism	↑ 91.5-fold	─	↑ 1866.8-fold	─	↑ 73.7-fold	─

*CYP1B1*	Xenobiotic metabolism	↑ 19-fold	↑ 2.7-fold	↑ 40.4-fold	↑ 3.2-fold	↑ 21.9-fold	↑ 2.3-fold

*GDF15*	Cell differentiation	↑ 11.2-fold	─	↑ 22.5-fold	↑ 8.9-fold	↑ 2-fold	─

*TIPARP*	PARP family	↑ 5.5-fold	─	↑ 5.8-fold	↑ 2.9-fold	↑ 6-fold	↑ 4.7-fold

*JUN*	Cell proliferation	↑ 5.5-fold	↑ 1.8-fold	↑ 5.1-fold	↑ 3.6-fold	─	─

*p21*	cell cycle checkpoints	↑ 3.2-fold	─	↑ 6.6-fold	─	─	─

*RASAL1*	Ras regulator	↑ 3.4-fold	─	↑ 2.8-fold	↑ 3.3-fold	─	↓ 2.8-fold

*RGC32*	Cell cycle control	↑ 2.9-fold	↑ 1.6-fold	↑ 2.3-fold	─	↑ 2.3-fold	↑ 1.5-fold

*ALDH1A3*	Xenobiotic metabolism	↑ 1.7-fold	─	↑ 4.2-fold	↑ 4.3-fold	↑ 2.4-fold	─

*DLX2*	Development	↑ 1.5-fold	─	↑ 4.8-fold	↑ 5-fold	↑ 2.5-fold	↑ 3.3-fold

*CEBPA*	Mitotic growth arrest	↑ 1.7-fold	─	─	─	↑ 1.5-fold	↑ 1.7-fold

*NRF2*	Oxidative stress	↑ 1.5-fold	─	─	─	↑ 1.6-fold	↑ 1.6-fold

*ATF3*	Transcription Factor	↑ 3-fold	─	↑ 6.1-fold	↑ 4.5-fold	─	

*DNAJB4*	Oxidative stress	↑ 2.2-fold	─	↑ 1.6-fold	↑ 1.9-fold	─	↑ 1.5-fold

*Bax*	Apoptosis	↑ 1.5-fold	─	↑ 1.6-fold	─	─	↑ 1.6-fold

*SPRY4*	MAPK signalling	─	─	─	↑ 4.5-fold	↑ 1.7-fold	─

*GSTT2*	Xenobiotic metabolism	↑ 2.8-fold	↑ 1.7-fold	─	─	─	─

*CABLES2*	Apoptosis	─	─	─	─	─	↑ 2.3-fold

*PTGER4*	T-cell factor signalling	─	↑ 2.7-fold	↑ 1.5-fold	─	─	↑ 2.2-fold

*BBC3*	Apoptosis	─	─	↑ 2.1-fold	↑ 5-fold	─	↑ 1.9-fold

*EGR1*	Cell growth and proliferation	↑ 6.7-fold	↑ 2.7-fold	↑ 2.5-fold	─	─	─

*CTNNB1*	Cell adhesion	↑ 1.5-fold	↑ 2.25-fold	─	─	─	─

*PLK3*	Cell cycle control	─	─	↑ 2.5-fold	↑ 3.4-fold	─	─

*AFF4*	Transcription Elongation	─	─	─	↓ 2.6-fold	↓ 1.5-fold	↓ 6.2-fold

*ZBRK1*	DNA damage response	─	─	─	↓ 4.7-fold	─	─

*DKK1*	Wnt/Catenin	↓ 7-fold	↓ 3.1-fold	↓ 2.8-fold	─	↓ 2.4-fold	─

*KAT2B*	Transcription regulation	↓ 4.6-fold	─	↓ 3-fold	─	↓ 6.5-fold	↓ 1.7-fold

*RASGRP1*	Ras signalling	↓ 3.8-fold	↓ 1.5-fold	↓ 2.2-fold	─	↓ 6-fold	↓ 2-fold

*JMJD2C*	Histone demethylase	↓ 3.6-fold	─	↓ 4.5-fold	↓ 2-fold	↓ 3-fold	↓ 1.8-fold

*HDAC4*	Cell cycle control	↓ 2.1-fold	─	↓ 2.7-fold	↓ 1.8-fold	↓ 1.8-fold	─

*FERMT1*	Actin cytoskeleton	↓ 2.1-fold	↓ 6.7-fold	↓ 1.8-fold	─	↓ 2.5-fold	─

*Scaper*	cell cycle checkpoints	↓ 1.8-fold	↓ 1.5-fold	↓ 1.8-fold	↓ 2-fold	↓ 2.8-fold	↓ 2.2-fold

*NPM1*	Cell proliferation/Apoptosis	─	─	─	─	─	↑ 2.4-fold

*YWHAQ*	Cell cycle control/Apoptosis	─	─	─	─	─	─

*AHR*	Xenobiotic Metabolism	─	─	─	─	─	─

*p53*	Tumour Suppressor	─	─	─	─	─	─

*RFC5*	Replication Factor	─	↑ 1.8-fold	─	─	─	─

*Hint1*	Apoptosis	─	↑ 2.2-fold	─	─	─	↑ 1.5-fold

*NQO1*	Xenobiotic metabolism	─	↑ 2.2-fold	─	─	─	─

A 1.5-fold cut-off was used. ↑ denotes up-regulation, and ↓ denotes down-regulation.

### Protein expression

**Figure 9:**
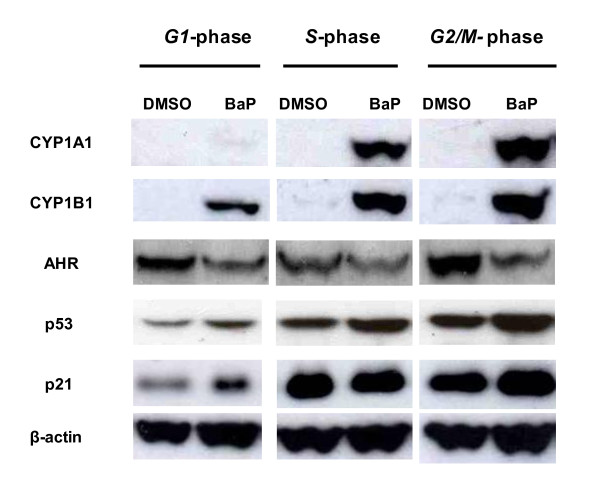
**Expression of CYP1A1, CYP1B1, AHR, p53, and p21 protein levels in MCF7 cells in different phases of the cell cycle in response to BaP detected by Western blots**. Cells were treated with DMSO (0.3%) or BaP (2.5 μM) for 12 h. 15 μg protein was loaded in each lane. β-actin was used as a loading control.

There was a clear induction of both CYP1A1 and CYP1B1 proteins after BaP exposure in all phases, but to a greater extent in *S- *and *G2/M- *than in *G1-*enriched cultures (Figure 9). Band quantification showed that there was a 1.5-fold higher level of CYP1B1 in *S- *and *G2/M- *than in *G1-*enriched cultures after BaP treatment. Similarly, the amount of CYP1A1 protein after BaP exposure was 5 to 6-fold higher in *S*- and *G2/M*- than in *G1-*enriched cultures. These findings correlate strongly with levels of DNA adducts seen in the different phases. There was a down-regulation of AHR after BaP treatment, as the protein levels were lower by 2-fold in BaP-treated compared to DMSO control cells in all enriched cultures.

A number of *TP53*-regulated genes were modulated in response to BaP exposure at: a) the microarray level: *STMN1 *in *G1 *only; *GDF15 *and *BTG2 *in *S *only; *PCAF, BAX, SESN1, ASPM, MBNL2, CABLES2 *and *Scaper *in *G2/M *only; *c-Jun *and *BTG3 *in *G1 *and *S*; *HINT1 *and *RGC32 *in *G1 *and *G2/M*; b) the RT-PCR level: *CDKN1A, GDF15*, and *RGC32 *in all phases. Other genes that regulate *TP53 *activity, such as *MDM4 *and *NPM1*, were also modulated by BaP.

However, as expected, induction of *TP53 *gene expression was not observed on the microarrays and this was confirmed by RT-PCR. Therefore, p53 protein levels were assessed by Western blotting in order to confirm accumulation of this tumour suppressor in response to the BaP in different phases of the cell cycle (Figure 9). An increase in p53 protein was observed in MCF-7 cells after exposure to BaP in all phases with considerably more protein in *G2/M-*enriched cultures, underlying its significant role in the *G2/M *checkpoint. These profiles of p53 protein activation are similar to those of its direct target CDKN1A (p21), except that there was no induction in *S*-enriched cultures (Figure 9).

## Discussion

Microarray technology is a powerful tool for identifying gene expression patterns that are reflective of the response of cells to carcinogen exposure [23-25], and can be informative of mechanisms of action [26,27]. Using this technology we have investigated whether human cells are more susceptible to the environmental carcinogen BaP at particular phases of the cell cycle and, if so, to elucidate the mechanisms involved. The resulting gene expression profiles were related to other phenotypic measures of BaP exposure such as DNA damage and cell cycle distribution to further our biological understanding of BaP carcinogenesis.

We found that BaP induced more DNA damage in synchronised MCF-7 cells enriched in *S- *and *G2/M-*phases than in *G1*, which indicates that BaP is metabolised with varying efficiency at different stages of the cell cycle. This conclusion is supported by the fact that DNA damage induced by BPDE -which modifies DNA without further metabolism-, was not cell cycle dependent. Gene expression profiling data (mRNA) and protein expression of xenobiotic metabolising enzymes further supported this hypothesis. Indeed, *CYP1A1 *mRNA measured by RT-PCR was almost 20-fold higher in *S*-phase than in *G1 *and *G2/M*. *CYP1B1 *mRNA followed the same pattern with a 2-fold higher induction in *S*-phase. Moreover, comparison between the levels of *CYP1A1 *in different phases revealed more mRNA in *S*- and *G2/M-*enriched cultures. The same pattern was observed at the protein level for CYP1A1 and CYP1B1. These findings correlate well with the levels of BaP-DNA adducts at each phase of the cell cycle, consistent with the role of CYP1A1 and CYP1B1 in the metabolic activation of BaP to BPDE.

Previously, Jiao et al. [11] reported an up-regulation of *CYP1A1 *mRNA of > 100-fold in BaP-treated *S*-enriched MCF-7 cultures, while up-regulation in *G1- *and *G2/M-*enriched cultures occurred to a significantly lesser extent. Consistent with this, they also reported higher levels of DNA damage in the form of DNA single strand breaks and BaP-DNA adducts in *S*-enriched cultures. However, it is not the extent of induction that matters but the actual levels (absolute amounts) of mRNA and protein, because an apparently very strong induction could be due simply to very low basal levels. Moreover, Jiao and coworkers' BaP treatment was for 24 h, a duration that is long enough for the cells to progress to another phase of the cell cycle. In another study, Santini et al. [13] showed that within 3 h of TCDD exposure late *G1*/early *S-*phase U937 human leukaemic monocyte lymphoma cells had 1.4- and 3-fold higher *CYP1A1 *mRNA levels than asynchronous/early *G1 *and *G2/M *cultures, respectively. In contrast, we found that the absolute mRNA levels of *CYP1A1 *and the protein levels of both CYP1A1 and CYP1B1 were actually higher in *S*- and *G2/M*-enriched cultures. AHR down-regulation at the protein level in all enriched cultures was the result of suppressing AHR signalling by rapid proteosomal degradation. Ligand-dependent receptor activation is well established, and several mechanisms capable of suppressing prolonged AHR signalling have been identified. These include rapid receptor degradation, the action of an AHR Repressor protein (AHRR), and agonist depletion through an enzymatic negative feedback loop [28]. AHR activation independent of agonist binding in mammalian systems has been proposed, but evidence for it is not yet conclusive. Chang and Puga [29] reported that AHR-dependent effects on cell proliferation could be dissociated from exogenous ligand binding. In another study, cell density, but not cell cycle, was shown to influence the intracellular distribution of AHR [30]. However, neither study established the absence of an endogenous ligand responsible for receptor activity.

Preliminary experiments have indicated that there are no differences in BaP-DNA adduct formation between *G0- *and *G1-*enriched MCF-7 cultures (data not shown). Therefore, adduct levels in *G0- *are lower than *S*- and *G2/M*-enriched cultures. *G0 *cells are quiescent *i.e*. not cycling, as is the case with many cell types in mammalian tissues. Interpreting the adduct data in the light of this information could point to a difference in susceptibility to genotoxic carcinogens between proliferating (cycling cells that go beyond *G1 *to *S*, and *G2/M*) and non-proliferating cells (mainly in *G0*).

BaP exposure resulted in an arrest of the cells in *S-*phase of the cell cycle in *S- *and *G2/M-*enriched cultures, indicating that interruption of DNA synthesis had occurred. This is in agreement with other studies that have shown the inhibition of DNA synthesis in response to BaP [9,31]. The pause in DNA synthesis is probably due to the intra-*S *checkpoint, which allows repair enzymes time to recognize the damaged DNA and to correct it, avoiding irreversible errors during replication (i.e. mutations). Alternatively, a permanent growth arrest or apoptosis can be initiated if damage is too great or persists for too long [32].

We found that BaP did not activate the *G1/S *checkpoint despite p53 and p21 protein induction in these phases. The *G1 *arrest delays DNA damaged cells from progressing through the cell cycle, avoiding accumulation of mutations and chromosomal aberrations by means of DNA repair or apoptosis. *TP53 *and its transcriptional target *CDKN1A *(*p21*) contribute to *G1 *and *G2 *arrest in response to DNA damage to maintain genomic stability [33]. These responses consist of the ATM(ATR)/CHK2(CHK1)-p53/MDM2-p21 pathway, which is capable of sustaining *G1 *arrest. Phosphorylation of p53 transcription factor and MDM2 (which normally binds to p53 and ensures rapid p53 turnover) results in p53 stabilisation and accumulation. p21, in turn, inhibits cyclin E(A)/CDK2 and preserves the RB/E2F pathway in its active, growth-suppressing mode [32,34,35].

In one study, Khan and Dipple [7] showed that following treatment with a range of agents, including metabolites of BaP, *G1 *arrest does not occur in MCF-7 cells and other cell lines. They also demonstrated that BPDE is not effective in arresting MCF-7 cells in *G1 *in spite of inducing dose-dependent increases in p53 and p21 [36]. The ability of carcinogens to induce cells to evade the *G1 *DNA-damage checkpoint and progress into *S*-phase is known as the stealth property. This property presumably enhances the mutation frequency and increases the likelihood of malignant changes.

In another study, Jiao et al. [37] investigated the mechanisms by which BaP accelerates cell cycle progression (*G1/S *transition) and induces cell proliferation in human embryo lung fibroblasts. They also found that c-Jun activation (by phosphorylation) by p53-dependent PI-3K/Akt/ERK pathway might be responsible for BaP-induced cell cycle alterations. Interestingly, *JUN *mRNA was up-regulated by BaP in our study in both *G1*- and *S*-enriched cultures. In addition to that, our pathway analysis showed it to be significantly involved in Network 5B (*G1*-enriched cells) and Network 6A (*S*-enriched cells).

Gene Ontology analysis revealed several over-represented biological themes after BaP exposure. These include cell differentiation, cell proliferation, cell cycle regulation and xenobiotic metabolism. In *G1*-enriched cultures, some modulated genes belonged to cell differentiation (11%) and cell proliferation (8%) functional groups. One of these genes is *BTG3 *(up-regulated), which has been identified as a DNA damage-inducible *CHK1*-modulated gene. As it is a direct p53 target this emphasises its importance in cell cycle regulation and in maintaining genome stability [38]. Another example of modulated genes involved in regulating cell proliferation and differentiation is *EGR1*, which was also revealed by pathway analysis (Network 5B in *G1*-enriched cells). Modulation of the expression of this gene was validated by RT-PCR and it was shown to be induced in *G1*-, and *S*-enriched cultures. Several xenobiotic metabolism genes were also modulated by BaP, including *CYP1B1, GSTT2 and NQO1*. Detoxification of PAH quinone metabolites is carried out by NAD(P)H:quinone oxidoreductase encoded by *NQO1 *[39], which is also required for p53 stabilisation in response to DNA damage [40]. Glutathione S-transferase T2 (GSTT2) is involved in cellular defence against toxic and carcinogenic electrophilic compounds by conjugation of reduced glutathione to hydrophobic electrophiles [41], so it was a logical finding that *GSTT2 *was up-regulated in response to BaP exposure.

Pathway analysis revealed the activation of the Catenin/Wnt pathway (Network 5A) in the response to BaP exposure. Consistent with this, RT-PCR analysis showed that *DKK1 *(a Wnt antagonist) was down-regulated (by 7-fold) in *G1*-enriched cultures and *CTNNB1 *was up-regulated (by 1.4-fold) in the same cultures.

In *S*-phase, cell proliferation and apoptosis genes such as *BTG2 *and *HDAC4 *were also differentially expressed. Previously, our team showed that *BTG2 *was up-regulated by BaP and BPDE at different time points in MCF-7 cells [9,10]. Its expression was also shown to be induced by genotoxic stress through a p53-dependent mechanism [42]. *HDAC4*, which encodes a histone deacetylase that represses transcription and regulates differentiation, was down-regulated in our experiments [43]. Differentially expressed genes involved in PAH metabolism included *CYP1B1*, *AKR1C1, ALDH1A3 *and *UGT1A6*. *CYP1B1 *(also induced in *G1*- and *G2*/*M*-enriched cultures) encodes a member of the CYP superfamily of monooxygenases and is involved in the metabolic activation of BaP [44,45]. Interestingly, enhanced expression of this enzyme has been observed in a number of cancers [46,47] and it has been demonstrated, in experiments involving CYP1B1-null mice, that it enhances the carcinogenicity of 7,12-dimethylbenz[a]anthracene [48]. *CYP1B1 *has also been found to be up-regulated in primary human mammary epithelial cells exposed to BaP, highlighting the importance of this enzyme in BaP metabolism in this tissue [49]. Consistent with previous studies [9,50], *AKR1C1 *was also found to be up-regulated by BaP. It encodes an aldo-keto reductase capable of metabolising PAH trans-dihydrodiols to *o*-quinones that can lead to the formation of DNA adducts and reactive oxygen species (ROS) [50,51], thus providing another pathway of PAH genotoxicity. *UGT1A6 *is involved in glucuronidation, which is a major pathway for detoxification of PAH metabolites [52].

Another interesting gene function category revealed by the transcriptomic analysis was that of DNA-damage induced protein phosphorylation, as exemplified by *MAP2K6*. This gene encodes a member of the dual specificity protein kinase family, which functions as a mitogen-activated protein (MAP) kinase kinase. MAP kinases, also known as extracellular signal-regulated kinases (ERKs), act as an integration point for multiple biochemical signals. This protein phosphorylates and activates p38 MAP kinase in response to inflammatory cytokines or environmental stress. As an essential component of the p38 MAP kinase signal transduction pathway, *MAP2K6 *is involved in many cellular processes such as stress-induced cell cycle arrest, transcription activation and apoptosis [53].

In *G2/M*-phase, BaP altered the expression of several cell cycle regulation genes, including *NPM1, PCAF, NBN, RGC32, SESN1 *and *BAX *as shown by Gene Ontology and pathway analysis (Table 1 and **Network 7C**)*. NPM1 *(up-regulated) has been shown to be implicated in human tumourigenesis, functioning both as an oncogene and as a tumour-suppressor. It is involved in many pathways such as cell cycle control, DNA repair and apoptotic response to stress by modulating the activity and stability of critical tumour-suppressor proteins such as p53 [54]. *NBN *(up-regulated) is involved in cell cycle checkpoints in response to DNA damage [55]. *RGC32, SESN1 *and *BAX *(all up-regulated) are all targets of p53 contributing to its role in cell cycle regulation, metabolism and apoptosis [56-58]. Indeed, accumulation of p53 was seen after BaP treatment by Western blotting (Figure 9).

## Conclusions

Exposure of synchronized MCF-7 cells to BaP has identified a complex gene expression response by microarray analysis. A number of genes were found to have their expression altered by BaP, including those involved in xenobiotic metabolism, apoptosis, cell cycle regulation and DNA repair. Gene ontology and pathway analysis showed the involvement of various signalling pathways in the response to BaP, such as Catenin/Wnt pathway in *G1*, ERK pathway in *G1 *and *S*, Nrf2 pathway in *S *and *G2/M *and Akt pathway in *G2/M*.

A key finding in this study was that higher levels of DNA adducts in *S- *and *G2/M-*enriched cultures correlated with higher levels of *CYP1A1 *and *CYP1B1 *mRNA and protein expression, indicating that proliferating cells are more prone to DNA damage by genotoxic stress than non-proliferating cells. Our results clearly demonstrate that this is due to the varying efficiency of BaP metabolism through the cell cycle. Additional studies with other cells lines and genotoxic agents will be required to determine whether our findings, in terms of adduct formation and expression of CYP1A1 and CYP1B1 (both at the mRNA and protein level) are universal or specific to certain cell types.

## Methods

### Cell culture and treatment

MCF-7 human breast carcinoma cells were purchased from the European Collection of Cell Cultures (ECACC, Salisbury, UK). Cells were grown as adherent monolayers and maintained in Dulbecco's modified Eagle's medium with Glutamax™ I, 1000 mg/L D-glucose and sodium pyruvate (Invitrogen, Paisley, UK) and supplemented with 10% heat-inactivated foetal bovine serum (Invitrogen) and 100 U/mL penicillin and 100 μg/mL streptomycin (Sigma-Aldrich, Dorset, UK). Cells were incubated in a humidified 5% CO_2 _atmosphere at 37°C and sub-cultured every 72 h when the cells were 80% confluent.

Culture conditions were manipulated in order to generate (1) *G0/G1*-enriched cultures by serum deprivation for 48 h (cells were kept in *G0 *by not adding the serum back during the BaP treatement in contrast to *G1*-enriched cells); (2) *S*-enriched cultures by serum deprivation for 48 h followed by 18 h growth in complete media; and (3) *G2/M*-enriched cultures by treatment for 24 h with 1 μg/mL aphidicolin followed by 0.25 μM colchicine for 12 h. Cell cycle distributions, determined by flow cytometry are shown in Table 3.

**Table 3:** Cell cycle distribution of MCF-7 cells prior to BaP treatment

		Cell cycle distribution (%)		
**Culture manipulations (time, h)**		**G0/G1-phase**	**S-phase**	**G2/M-phase**

*G0/G1*-enriched	48 h serum deprivation	65.3 ± 2.1	14.1 ± 2.4	20.6 ± 2.4

*S*-enriched	18 h after 48 h serum deprivation	24.7 ± 4.2	55.7 ± 8.2	19.5 ± 4.0

*G2/M*-enriched	12 h colchicine (0.25 μM) after 24 h aphidicolin (1 μg/mL)	8.9 ± 2.2	18.2 ± 4.1	72.9 ± 5.9

Cells were manipulated to generate: *G0/G1*-, *S*-, and *G2/M*-enriched cultures.

Cells were seeded at 2 × 10^5 ^cells/ml and treated with BaP (2.5 μM), and BPDE (0.5 μM) for 12 hours. DMSO only was added to control cultures and its volume was kept at 0.3% of the total culture volume. Cells were harvested by trypsinisation followed by washing with PBS. All cell incubations for the different experimental applications were carried out in duplicate or triplicate.

### Flow cytometry

Harvested cells were re-suspended in 0.2 mL 10X PBS solution and fixed in 2 mL of ice-cold 70% ethanol. Samples were then stored at -20°C overnight. Twenty four hours prior to flow cytometry analysis, samples were centrifuged at 1500 × g for 5 minutes and resuspended in staining buffer containing 40 μg/mL propidium iodide (Invitrogen), 100 μg/mL RNase (Qiagen, UK) in PBS buffer at a final density of 1 × 10^6 ^cells/mL. Cells were then incubated at 37°C for 60 minutes and stored at 4°C overnight. The DNA content of 10,000 events per sample was analysed using a Beckman Coulter EPICS Elite ESP (Beckman Coulter, Buckinghamshire, UK) at 488 nm. The percentage of cells in each phase of the cell cycle was determined using Cylchred v1.0.2 and WinMDI v2.8 software [59]. Differences between control and treated cells were examined for statistical significance using Student's *t*-test (two-tailed).

### Cell viability

Cell viability (% control) was determined by cell counting with the CASY Model TT Electronic Cell Analyser (Innovatis AG, Germany).

### DNA adduct analysis

DNA was isolated from cell pellets by a standard phenol chloroform extraction method. DNA was quantified spectrophotometrically and DNA adducts were determined for each DNA sample using the nuclease P1 enrichment version of ^32^P-postlabelling method [60]. Briefly, DNA samples (4 μg) were digested with micrococcal nuclease (120 mU, Sigma, UK) and calf spleen phosphodiesterase (40 mU, Calbiochem, UK), then enriched and labelled as reported. Solvent conditions for the resolution of ^32^P-labelled adducts on polyethyleneimine-cellulose thin-layer chromatography (TLC) were as described [9]. After chromatography TLC plates were scanned using a Packard Instant Imager (Dowers Grove, IL, USA) and DNA adduct levels (RAL, relative adduct labelling) were calculated from the adduct cpm, the specific activity of [γ-^32^P]ATP and the amount of DNA (pmol of DNA-P) used. Results were expressed as DNA adducts/10^8 ^nucleotides. An external BPDE-DNA standard [61] was employed for identification of adducts in experimental samples.

### RNA isolation and whole-genome gene expression profiling

Total RNA was extracted from cells using the Qiagen RNeasy Mini Kit protocol (RNeasy Mini Handbook, Qiagen, UK). RNA was quantified spectrophotometrically, and integrity was determined using a 2100 Bioanalyser (Agilent Technologies, UK). Only RNA with an integrity number ≥ 9 was used for gene expression analysis.

Gene expression analysis was carried out using the Agilent two-colour microarray-based gene expression analysis (Agilent Technologies, UK), which uses cyanine 3 (Cy3)- and cyanine 5 (Cy5)-labelled targets to measure gene expression in experimental and control samples.

Agilent Human 4 × 44K Genome-wide arrays were used and the reference design was applied, whereby a Universal Human Reference RNA (Stratagene, La Jolla, USA) was hybridised to every sample. Cy3-(sample)- and Cy5-(reference)-labelled probes were hybridised to the oligo microarrays using the Gene Expression Hybridization Kit (Agilent Technologies, UK) using Agilent SureHyb chambers for 17 hours in Rotisserie Hyb Oven set to 65°C (Agilent) and rotating at 10 rpm. The array slides were washed according to the manufacturer's instructions (Agilent) and dried with compressed air prior to scanning on an Axon B400 Scanner (Axon, Instruments, USA).

### Microarray data analysis

The multi-image TIFF files generated by the scanner were exported to BlueFuse software, which adjusts the initial grid position and optimises spot finding in the image automatically so that each spot on the array is assigned a specific gene. BlueFuse software generated Excel files, which were analysed using GeneSpring v7.2 software (Silicon Genetics).

Data were imported into GeneSpring software and subjected to Per chip and Per spot lowess normalisation. Bad spots that were flagged in BlueFuse software were filtered out in order to give a gene list of reliable data. Cy3/Cy5 ratios of the 3 biological replicates were averaged and then used to identify modulated genes using 1 Way-ANOVA with a cut-off of 1.5-fold change and a Student's *t*-test *p-*value of less than 0.05. Over-representation analysis of differentially expressed genes was carried out using the Gene Ontology function within GeneSpring and Ingenuity pathway software.

The gene expression data discussed in this publication have been deposited in NCBI Gene Expression Omnibus [62] and are accessible through GEO Series accession number GSE26917.

### Real-time quantitative PCR

Two-step reverse transcription-PCR was used to generate cDNA for relative quantitation analysis using real-time fluorescent PCR. cDNA was reversed transcribed from 1 μg total RNA using random primers following the Superscript III Reverse Transcriptase First-Strand cDNA Synthesis Protocol (Invitrogen). cDNA was diluted 1:10 and 2 μL was used as template to perform RT-PCR in a 15 μL reaction. *GAPDH *was used as an endogenous control (Applied Biosystems, UK) in multiplexed PCR reactions on an ABI PRISM 7900HT Sequence Detection System (Applied Biosystems) with standard thermocycling conditions (50°C 2 min, 95°C 10 min, then 40 cycles of 95°C 15 s, 60°C 1 min), using Taqman Universal PCR Master Mix (Applied Biosystems). To confirm the modulated expression of the selected target genes, 20x Assays-On-Demand™ gene expression primers and probes (Applied Biosystems) were used. The list of the assays is provided as Additional file 8. Relative gene expression between the control (or calibrator) and treated samples was calculated after normalisation to the *GAPDH *reference using the comparative threshold cycle (C_T_) method.

### Western blot analysis

Cells were lysed in 800 μL lysis buffer (62.5 mM Tris pH 6.8, 1 mM EDTA pH 8.0, 2% SDS, 10% Glycerol). Samples were sonicated to break up the DNA and their protein concentration was determined using the BCA assay (Piercenet, UK) in order to load the same amounts of protein. Cell lysates were electrophoretically separated using Criterion XT 4-12% Bis-Tris gels (Bio-Rad, UK). Following electrophoresis, gels were transferred onto a nitrocellulose membrane (Millipore, UK). Ponceau staining was performed to check for the quality of transfer, and then the membranes were blocked by incubation in 5% non-fat dry milk dissolved in TBST overnight at 4°C. Blots were then incubated with primary antibody, thereafter with the species-specific horseradish peroxidase-conjugated secondary antibody and bands detected by chemiluminescence (ECL detection reagents, GE Healthcare). The following primary antibodies were purchases: anti-p53 (Ab-6; 1:2,000) from Calbiochem (Darmstadt, Germany), anti-p21 (BD 556431; 1:2,000) from BD Science (Oxford, UK), anti-CYP1B1 (CYP1B11-A; 1:2,000) from Alpha Diagnostic (Hampshire, UK), anti-AHR (Abcam ab2770; 1:1,000). Anti-CYP1A1 raised in rabbits against purified human recombinant CYP1A1 was a generous gift from F. Peter Guengerich (Vanderbilt University, USA) and was diluted 1:4,000. The antibody to detect β-actin (Ab6276; 1:25,000) was purchased from Abcam and used as a loading control. Two secondary horseradish peroxidise-linked antibodies were purchased: anti-mouse (CST 7076; 1:10,000), anti-rabbit (CST 7074; 1:10,000) from Cell Signalling Technologies (Herts, UK). Band quantification was carried out using ImageJ software [63].

## Authors' contributions

DP conceived the study and supervised its design and coordination. HH, IG and VA participated in design and coordination of the study. HH and IG designed the microarray experiments. HH carried out all experiments and data analysis with the exception of DNA adduct measurements, which were performed by VA. HH drafted the manuscript and DP, VA and IG participated in its preparation. All authors have read and approved the final manuscript.

## Supplementary Material

Additional file 1 **BaP delays escape from S-phase (Histogram representation)**. **A**- MCF-7 cells were synchronised in *G0/G1*-phase by serum-deprivation for 48 h, after which cells were exposed for 12 h to BaP (2.5 μM) or DMSO. **B**- MCF-7 cells were enriched in *S*-phase by serum-deprivation for 48 h, then left to grow for 18 h, after which they were treated by either BaP (2.5 μM) or DMSO for 12 h. **C**- MCF-7 cells were synchronised in *G2/M*-phase by exposing them to aphidicolin (1 μg/mL) for 24 h followed by colchicine (0.25 μM) for 12 h. Subsequently, they were released into media containing either BaP (2.5 μM) or DMSO for 12 h. Cell cycle distribution was examined by flow cytometry and DNA histograms were generated. The profiles are representative of three independent experiments. Click here for file

Additional file 2 **List of differentially-expressed genes common to *G1-*, *S- *and *G2/M*-enriched cultures after 12h BaP (2.5 μM) treatment**. Only genes which had a change of 1.5-fold after BaP exposure are shown. Click here for file

Additional file 3 **List of differentially-expressed genes common to *G1- *and *S-*enriched cultures only after 12h BaP (2.5 μM) treatment**. Only genes which had a change of 1.5-fold after BaP exposure are shown. Click here for file

Additional file 4 **List of differentially-expressed genes common to *G1- *and *G2/M*-enriched cultures only after 12h BaP (2.5 μM) treatment**. Only genes which had a change of 1.5-fold after BaP exposure are shown. Click here for file

Additional file 5 **List of differentially-expressed genes common to *S- *and *G2/M*-enriched cultures only after 12h BaP (2.5 μM) treatment**. Only genes which had a change of 1.5-fold after BaP exposure are shown. Click here for file

Additional file 6 **Ingenuity pathway analysis (IPA) figures legend**. Click here for file

Additional file 7 **List of the top five scoring network in each enriched culture**. Scores are obtained within Ingenuity pathway analysis (IPA) software. Click here for file

Additional file 8 **Gene expression primers and probes used in RT-PCR reactions**. The assays were purchased from Applied Biosystems and each consists of 2 primers (forward and reverse) and a Taqman probe. Click here for file
