# Supplementary figures and images for: Influence of cell cycle on responses of MCF-7 cells to benzo[a]pyrene
Source: BMC Genomics. 2011 Jun 29;12:333. doi: 10.1186/1471-2164-12-333 (PMC3145607; doi:10.1186/1471-2164-12-333)

**A**

Before BaP treatment


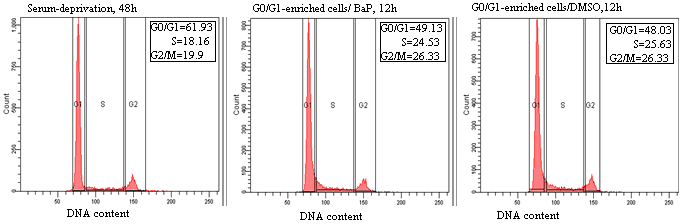


**B**

Before BaP treatment

**
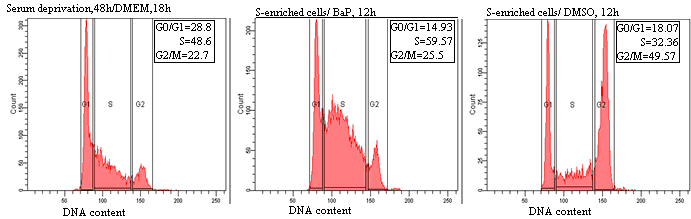
**

**C**

Before BaP treatment

**
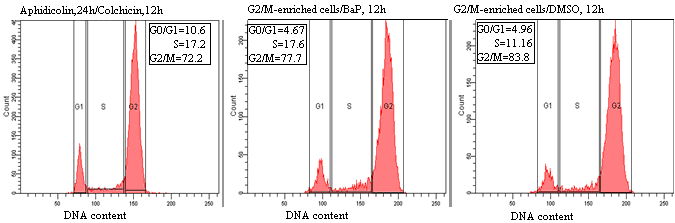
**

Supplement: Additional file 1 — BaP delays escape from S-phase (Histogram representation). A- MCF-7 cells were synchronised in G0/G1-phase by serum-deprivation for 48 h, after which cells were exposed for 12 h to BaP (2.5 μM) or DMSO. B- MCF-7 cells were enriched in S-phase by serum-deprivation for 48 h, then left to grow for 18 h, after which they were treated by either BaP (2.5 μM) or DMSO for 12 h. C- MCF-7 cells were synchronised in G2/M-phase by exposing them to aphidicolin (1 μg/mL) for 24 h followed by colchicine (0.25 μM) for 12 h. Subsequently, they were released into media containing either BaP (2.5 μM) or DMSO for 12 h. Cell cycle distribution was examined by flow cytometry and DNA histograms were generated. The profiles are representative of three independent experiments. [file 1471-2164-12-333-S1.DOC]

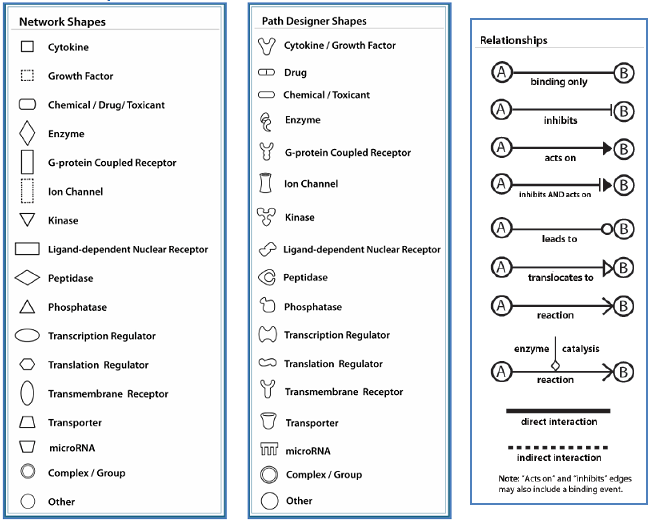

Supplement: Additional file 6 — Ingenuity pathway analysis (IPA) figures legend. [file 1471-2164-12-333-S6.DOC]
