# Supplementary material for: Influence of cell cycle on responses of MCF-7 cells to benzo[a]pyrene
Source: BMC Genomics. 2011 Jun 29;12:333. doi: 10.1186/1471-2164-12-333 (PMC3145607; doi:10.1186/1471-2164-12-333)
Supplement: Additional file 2 — List of differentially-expressed genes common to G1-, S- and G2/M-enriched cultures after 12h BaP (2.5 μM) treatment. Only genes which had a change of 1.5-fold after BaP exposure are shown. [file 1471-2164-12-333-S2.DOC]

| Agilent ID | Gene symbol |
| --- | --- |
| A_24_P68908 | LOC344887 |
| A_32_P118568 | RFPL1S |
| A_23_P358709 | AHRR |
| A_23_P209625 | CYP1B1 |
| A_23_P395438 | HTRA3 |
| A_32_P165477 | SLC7A11 |
| A_32_P104053 | FER |
| A_23_P2573 | TMEM117 |
| A_23_P148249 | THSD4 |
| A_23_P163408 | ZNF291 |
| A_23_P17503 | C20orf23 |
| A_23_P105862 | FRY |
| A_24_P324886 | DOCK4 |
| A_32_P50973 |  |
| A_23_P212688 | EVI1 |
| A_23_P422911 | HS6ST3 |
