# Supplementary material for: Influence of cell cycle on responses of MCF-7 cells to benzo[a]pyrene
Source: BMC Genomics. 2011 Jun 29;12:333. doi: 10.1186/1471-2164-12-333 (PMC3145607; doi:10.1186/1471-2164-12-333)
Supplement: Additional file 3 — List of differentially-expressed genes common to G1- and S-enriched cultures only after 12h BaP (2.5 μM) treatment. Only genes which had a change of 1.5-fold after BaP exposure are shown. [file 1471-2164-12-333-S3.DOC]

| Agilent ID | Gene Symbol |
| --- | --- |
| A_23_P201538 | JUN |
| A_23_P80068 | BTG3 |
| A_23_P167066 | UGDH |
| A_32_P167631 |  |
| A_23_P369479 | MSI2 |
| A_23_P211028 | NCAM2 |
| A_32_P217261 |  |
| A_23_P332399 | GULP1 |
| A_23_P85004 | DIAPH2 |
| A_23_P59637 | DOCK4 |
| A_24_P142495 | KRTAP1-3 |
