# Supplementary material for: Influence of cell cycle on responses of MCF-7 cells to benzo[a]pyrene
Source: BMC Genomics. 2011 Jun 29;12:333. doi: 10.1186/1471-2164-12-333 (PMC3145607; doi:10.1186/1471-2164-12-333)
Supplement: Additional file 4 — List of differentially-expressed genes common to G1- and G2/M-enriched cultures only after 12h BaP (2.5 μM) treatment. Only genes which had a change of 1.5-fold after BaP exposure are shown. [file 1471-2164-12-333-S4.DOC]

| Agilent ID | Gene Symbol |
| --- | --- |
| A_23_P136724 |  |
| A_32_P17343 |  |
| A_32_P42684 | SLC7A11 |
| A_23_P148047 | PTGER4 |
| A_23_P258944 | DNAJB9 |
| A_23_P83838 | CA8 |
| A_32_P47554 | HINT1 |
| A_24_P379413 | IL6R |
| A_32_P167000 |  |
| A_24_P566853 |  |
| A_23_P59613 | FZD9 |
| A_23_P157809 | LTB4DH |
| A_23_P204937 | C13orf15 |
| A_24_P924681 |  |
| A_23_P343671 | FOSL2 |
| A_24_P265856 | SENP7 |
| A_23_P124642 | RASGRP1 |
| A_32_P70245 |  |
| A_23_P12884 | GRK5 |
| A_24_P6944 | FAM128B |
| A_23_P157784 | HINT2 |
| A_23_P500936 | FOXA2 |
| A_23_P58148 | TMEM175 |
| A_23_P148990 | HMCN1 |
| A_23_P218346 | TANC2 |
| A_23_P367816 |  |
| A_32_P99100 | PTPRK |
| A_24_P255609 |  |
| A_23_P142537 | MBD5 |
| A_32_P27240 |  |
| A_24_P558141 |  |
| A_23_P375524 | LCE1D |
| A_24_P377775 | RGS3 |
| A_23_P207939 | C18orf1 |
| A_24_P205045 | ERC2 |
| A_24_P15640 | C19orf31 |
| A_24_P923514 |  |
