# Supplementary material for: Influence of cell cycle on responses of MCF-7 cells to benzo[a]pyrene
Source: BMC Genomics. 2011 Jun 29;12:333. doi: 10.1186/1471-2164-12-333 (PMC3145607; doi:10.1186/1471-2164-12-333)
Supplement: Additional file 5 — List of differentially-expressed genes common to S- and G2/M-enriched cultures only after 12h BaP (2.5 μM) treatment. Only genes which had a change of 1.5-fold after BaP exposure are shown. [file 1471-2164-12-333-S5.DOC]

| Agilent ID | Gene Symbol |
| --- | --- |
| A_23_P28595 | DLX2 |
| A_23_P28598 | DLX2 |
| A_23_P166109 | FLRT3 |
| A_23_P139600 | RASAL1 |
| A_23_P143845 | TIPARP |
| A_32_P4882 |  |
| A_23_P344400 | AHRR |
| A_32_P38093 |  |
| A_24_P393958 | DNAJB4 |
| A_23_P75609 | CEP164 |
| A_23_P6674 | LXN |
| A_23_P70998 |  |
| A_24_P100551 | SH3RF1 |
| A_23_P200325 | RABGAP1L |
| A_24_P333525 | RABGAP1L |
| A_23_P207445 | MAP2K6 |
| A_23_P92042 | ITPR1 |
| A_23_P79978 | SLC24A3 |
| A_23_P111531 | GLI3 |
| A_24_P331882 |  |
| A_32_P114284 | IKZF2 |
| A_23_P26037 | FRMD5 |
| A_32_P38623 | PPP1R9A |
| A_23_P110624 | CTNND2 |
| A_23_P112201 | JMJD2C |
| A_23_P334883 | SHANK2 |
| A_23_P119794 |  |
| A_24_P942636 | AFF4 |
| A_23_P375354 | TECTA |
| A_24_P38316 |  |
| A_24_P8130 |  |
| A_24_P130363 | C18orf1 |
