# Supplementary material for: Influence of cell cycle on responses of MCF-7 cells to benzo[a]pyrene
Source: BMC Genomics. 2011 Jun 29;12:333. doi: 10.1186/1471-2164-12-333 (PMC3145607; doi:10.1186/1471-2164-12-333)
Supplement: Additional file 7 — List of the top five scoring network in each enriched culture. Scores are obtained within Ingenuity pathway analysis (IPA) software. [file 1471-2164-12-333-S7.DOC]

| **ID** | **Molecules in Network** | **Score** | **Focus Molecules** | **Top Functions** |
| --- | --- | --- | --- | --- |
| ***G1*-enriched cultures** | | | | |
| 1 | Akt, APP, ARIH2, ATF4, BCL2L11, BIRC2, BLMH, Caspase, CDH10, CHD8, COX7A2, CTNNB1, Cytochrome c oxidase, DFNA5, DKK1, Frizzled, FSH, FZD7, KRT18, MCL1, MTTP, NKX3-1, PHIP, PKD2, (includes EG:5311), PRDX3, PRKAR1A, Proteasome, PTN, PTPRK, RPRM, Tubilin, Vegf, Wnt, WNT14, WNT8B | 36 | 29 | Organismal Development, Cell Death, Connective Tissue Disorders |
| 2 | ANGPT2, Calpain, Cbp/p300, CD3, CD4, Creb, Cyclin A, EGR1, ERK, ERK1/2, Fgf, FGF13, FGFR2, FZD9, GNAS, HOXB7, HSPE1, IL27RA, JUN, MAP2K1/2, Mek, MHC Class II, Mhc ii, MYLK, NAB1, NF1, PAQR3, PAWR, Pkg, PTGER4, RASGRP1, RGS3, STAT, STMN1, TCR | 35 | 22 | Reproductive System Disease, Gene Expression, Skeletal and Muscular System Development and Function |
| 3 | Ap1, C10ORF10, CaMKII, CD40, CD1D, CSRP2, IFN Beta, Ifn gamma, Iga, Igm, Ikb, IL10, IL12 (complex), KYNU, MAP3K7IP3, MHC Class II, Mhc ii, MYLK, NF1, NFkB (complex), Nos, NR4A3, PAWR, Pdgf, peptidase, Pkg, RELT, RFC5, RGS3, SYT1, TXN, TXNRD1, VAV, WNT11, ZFAND5 | 28 | 19 | Cell-mediated Immune Response, Cellular Growth and Proliferation, Hematological System Development and Function |
| 4 | 1,4,5-IP3, ACPL2, ANAPC4, CA8, CA9, Ca2+, CDH1, EDN3, EHF, FBXL20, FBXO24, FCN2, FGF23, GALNT3, IL13, LMO7, LRCH1, MAPK3, MASP1, MET, MIR1-1 (includes EG:406904), NCALD, NFKBIA, PCM1, PHIP, phosphatidylinositol 4,5-diphosphate, PIR, PKD2 (includes EG:5311), RASGRP1, RCVRN, RGS1, RGS3, SKP1, SLC12A6, SYTL2 | 27 | 18 | Carbohydrate Metabolism, Lipid Metabolism, Small Molecule Biochemistry |
| 5 | AGR2, Ahr-Arnt, Ahr-aryl hydrocarbon-Arnt, AHRR, BAMBI, BTG3, CASP1, CNTN1, DHCR24, DOCK4, EVI1, GSTT2, KIAA1804, KIF16B, MIR124, MIR222 (includes EG:407007), MIR34A (includes EG:407040), MT1L, MTPN, PAPSS2, PECI, PSG9, PTPN12, retinoic acid, SCAPER, SCML1 (includes EG:6322), SENP2, SFRS2IP, SMARCA4, STARD10, SUMO1, THSD4, UBFD1, YWHAB, ZFP2 (includes EG:80108) | 22 | 16 | Cancer, Cellular Growth and Proliferation, Tumor Morphology |
| ***S*-enriched cultures** | | | | |
| 1 | ADCY6, AKAP13, ANKRA2, ATF3, BTG2, CA3, CREB5, CYP1B1, DIAPH2, DLX2, ERBB4, FBP1, GAD1, GAD2, GJA1, GJB3, GNAQ, HDAC4, HOXC9, IKZF2, IL23A, JUN, LIF, MAP2K6, MECOM, MT2A, PLK3, PRKCE, RASA3, S1PR2, S1PR3, SPRR1A, UXT, VRK1, WNT7A | 34 | 21 | Cellular Growth and Proliferation, Cellular Development, Tissue Morphology |
| 2 | ACO1, AKR1C1, ALDH1A3, ARHGAP18, ARVCF, ATF3, ATP1A3, ATXN1, BTG2, BTG3, Cbp/p300-Maf-Nfe2l2, CTNND2, ERBB2IP (includes EG:55914), FTL, GCLC, GDF15, Hydrogen peroxide, INPP5D, JINK1/2, JUN/JUNB/JUND, MAFF, MAPKAPK5, MPHOSPH6, MPP6, MSR1, NADH dehydrogenase, NDUFA11, NFE2L2, PDZD2, PKP4, PLK3, SLC7A11, Sod, TNF, UGT1A6 | 34 | 18 | Cell Signaling, Molecular Transport, Small Molecule Biochemistry |
| 3 | AKAP7, AMD1, APP, ARHGDIA, ARVCF, BANP, BTG3, CTBP1, DOCK4, EPB41L1, FKSG17, FOXP1, FOXP2, GRB2, HIF1A, HIPK2, HTT, MAP3K9, MAPK8, MEPCE, MIR214 (includes EG:406996), NDUFA11, NT5E, RHOU, RTN3, SCAPER, SENP2, SET, SETBP1, SH3RF1, SHANK2, SLC7A11, SUMO1, TMEM168, USP17L2 | 23 | 16 | Cellular Assembly and Organization, Cellular Compromise, Inflammatory Disease |
| 4 | AFF4, BIRC3, CALB1, CIRBP, DHFR, DIABLO, E2F7, FER (includes EG:2241), FN1, IFNG, IKBKG, KIF16B, KLF6, LATS1, LIMK1, LMCD1, MCM6, NCAM2, NOB1, NPY1R, OSBPL6, PCK1, PENK, PIAS4, PIP5K1B, PLK1, PRNP, RB1, SCNM1, SPHK1, TAB1, TAB3, TMF1, TRAF2, TYMS | 19 | 14 | Cell Death, Cell-To-Cell Signaling and Interaction, Cellular Growth and Proliferation |
| 5 | ARHGAP18, ARHGDIA, ASGR2, BMP6, COL8A1, CREB3, DAD1, DLX5, FBLN5, FN1, FOXC1, FTL, HOXA2, HSP90B1, ITGA5, ITGAV, ITGB8, MEIS2, MMP9, MPHOSPH6, MPP6, MSR1, NPAS2, PBX1, PDX1 (includes EG:3651), RABGAP1L, SERP1, ST8SIA1, STK17A, TGFB1, TXNDC5, UGDH, VTN, XBP1, ZNF350 | 18 | 13 | Cell-To-Cell Signaling and Interaction, Tissue Development, Skeletal and Muscular System Development and Function |
| ***G2/M*-concentrated cultures** | | | | |
| 1 | Akt, Alpha actin, Ap1, APBB2, APP, AUTS2 (includes EG:26053), BAX, BBC3, Caspase, CNOT1, COX6A2, CTGF, CYR61, Cytochrome c oxidase, DLD, EGR3, HINT1 (includes EG:3094), HLA-G, IFIT2, IFN Beta, IL1, Interpheron alpha, LDL, MEF2D, NBN, nCG, Nfat, OSBPL6, PDHA1 (includes EG:5160), PDK4, PRKCA, PRKCB, THOP1, TTC28, TXNIP | 35 | 26 | Lipid Metabolism, Small Molecule Biochemistry, Free Radical Scavenging |
| 2 | AHRR, Ahr-aryl hydrocarbon-Arnt, AKT2, Alcohol group acceptor phosphotransferase, C20ORF24, CARD6, DNAJA3, DNAJB4, DNAJB9, GOT2, GRK5, GST, GSTM1 (includes EG:2944), HSF1, Hsp22/Hsp40/Hsp90, Hsp27, Hsp70, Hsp90, MAP2K6, MAP3K5 (includes EG:4217), MKK3/6, NCF2, NFE2L2, NFKB, P2RX7, P38/MAPK, PGR, Proteasome, PTPLAD1, SH3RF1, SLC7A11, SWI-SNF, TLR2, Ubiquitin, Vegf | 30 | 25 | Gene Expression, Cellular Development, Cancer, oxidative stress |
| 3 | ALPP, C13ORF15, C20ORF24, CSF2RB, DLX2, DLX5, EGR3, ELL2, FOS, FOSL2, GAD1, GCLM, GCNT1, HSPA4L, IL5, KDELR2, KLF16, KRT8, KRT17, MAPK6, MBNL2, MMP10, NCF2, PDGFC, PRKCB, PTPRK, RBM3, RPS17 (includes EG:6218), SBF2, SLC7A5, SMOC2, SUPT6H, TGFB1, TGFBI, TUBA3D | 24 | 20 | Gene Expression, Connective Tissue Disorders, Genetic Disorder |
| 4 | AKT2, ANXA2, ASAP1, CALM3, EEF1D, FCHSD2, GLI1, GLI3, IGF1R, LXN, MAGEB2, MAN1C1, NIPAL2, PINX1, RPL22, RPL24, RPL26, RPL31, RPL32, RPL34, RPL35, RPL18A, RPL21 (includes EG:6144), RPL29 (includes EG:6159), RPL35A, RPL36 (includes EG:25873), RPL37A (includes EG:6168), RPS25, RPS26, SH3RF1, SRP14, SRP72, STK36, VAV3, ZIC1 | 20 | 18 | Protein Synthesis, Cellular Movement, Developmental Disorder |
| 5 | ADAMTS7, ANKRD29, ARHGAP18, ASPM, Basc, CABLES2, COMP, FANCI, GLYCTK, KIAA0406, MBNL2, MGMT, MLH1, MPHOSPH6, MRE11A, MSH2, MSH3, NBN, NCAPG (includes EG:64161), NPM1, PSD3, PTP4A3, RAD50, RECQL, S100B, SCAPER, SESN1, SLC2A1, SMPD1, SOD2, SUMO1, TP53, UBE2K, XPO7, Zn2+ | 19 | 16 | Cell cycle, Genetic Disorder, Cellular Function and Maintenance |
