# Supplementary material for: Influence of cell cycle on responses of MCF-7 cells to benzo[a]pyrene
Source: BMC Genomics. 2011 Jun 29;12:333. doi: 10.1186/1471-2164-12-333 (PMC3145607; doi:10.1186/1471-2164-12-333)
Supplement: Additional file 8 — Gene expression primers and probes used in RT-PCR reactions. The assays were purchased from Applied Biosystems and each consists of 2 primers (forward and reverse) and a Taqman probe. [file 1471-2164-12-333-S8.DOC]

| **Gene Name** | **RT-PCR assay ID** | **Gene Name** | **RT-PCR assay ID** |
| --- | --- | --- | --- |
| CYP1A1 | Hs00153120_m1 | BBC3 | Hs00248075_m1 |
| CYP1B1 | Hs00164383_m1 | EGR1 | Hs00152928_m1 |
| GDF15 | Hs00171132_m1 | CTNNB1 | Hs00170025_m1 |
| TIPARP | Hs00296054_m1 | PLK3 | Hs00177725_m1 |
| JUN | Hs00277190_s1 | AFF4 | Hs00232683_m1 |
| p21 | Hs00355782_m1 | ZBRK1 | Hs00222159_m1 |
| RASAL1 | Hs00183013_m1 | DKK1 | Hs00183740_m1 |
| RGC32 | Hs00204129_m1 | KAT2B | Hs00908805_m1 |
| ALDH1A3 | Hs00167476_m1 | RASGRP1 | Hs00996734_m1 |
| DLX2 | Hs00269993_m1 | JMJD2C | Hs00325678_m1 |
| CEBPA | Hs00269972_s1 | HDAC4 | Hs00195814_m1 |
| NFE2L2 | Hs00232352_m1 | FERMT1 | Hs00214355_m1 |
| ATF3 | Hs00231069_m1 | Scaper | Hs00363558_m1 |
| DNAJB4 | Hs00199826_m1 | AHR | Hs00169233_m1 |
| Bax | Hs00180269_m1 | p53 | Hs00153349_m1 |
| SPRY4 | Hs00229610_m1 | RFC5 | Hs00201278_m1 |
| GSTT2 | Hs00168315_m1 | Hint1 | Hs00602163_m1 |
| CABLES2 | Hs00292775_m1 | NQO1 | Hs00168547_m1 |
| PTGER4 | Hs00168761_m1 |  |  |
